# Can Breast Self-Examination Continue to Be Touted Justifiably as an Optional Practice?

**DOI:** 10.1155/2011/965464

**Published:** 2011-04-06

**Authors:** T. T. Fancher, J. A. Palesty, J. J. Paszkowiak, R. P. Kiran, A. D. Malkan, S. J. Dudrick

**Affiliations:** Stanley J. Dudrick Department of Surgery, St. Mary's Hospital, 56 Franklin Street, Waterbury, CT 06706, USA

## Abstract

In 2003, the revised American Cancer Society guidelines recommended that breast self-examination (BSE) be optional. Of 822 women diagnosed with breast cancer in our hospital from 1994 to 2004, sixty four (7.7%) were 40 years of age or younger. Forty four (68.7%) of these young women discovered their breast cancers on BSE, 17 (18%) by mammography, and 3 (4.7%) by clinical breast examination by medical professionals. Of 758 women over 40 years of age diagnosed with breast cancer, 382 (49%) discovered their cancer by mammography, 278 (39%) by BSE, and 98 (14%) by a clinical breast examination. Lymph node metastases in the older women was one-half that in the younger women (21% versus 42%), and a higher percentage of younger women presented with more advanced disease. In response to increasing breast cancer in young women under 41 years of age, encouragement of proper breast self-examination is warranted and should be advocated.

## 1. Introduction

Our interest in undertaking this study was influenced by the following disconcerting factors. Breast cancer is the most common malignancy in females, accounting for 1 of 4 cancers diagnosed, and it is the second leading cause of deaths due to cancer among North American women. The American Cancer Society predicted that cancer of the breast would be diagnosed in 254,650 women and that 40,170 women would die secondary to breast cancer in 2009 [1]. Moreover, the American Cancer Society estimated that 25,100 new breast cancers would be diagnosed, with 2,820 deaths secondary to breast cancer, occurring in American women under 45 years of age in 2009 [1]. The incidence of initial presentation of breast cancer is known to peak between the ages of 45 and 55 years, and although women 40 years of age and under account for less than 5% of those developing this disease, we observed that during the last ten years, the incidence of breast cancer has increased most markedly in this younger population. Changes in diet, an escalating incidence of obesity, and an increased exposure to endogenous and exogenous hormones may have contributed to this increase, together with the decisions by larger numbers of women in the developed world to delay their first pregnancy until later in life. Finally, the 5-year survival rate is lower for women diagnosed with breast cancer before age 40 (83%) compared with women diagnosed at ages 40 years or older (90%). These observations prompted us to examine the differences that might exist between young women 40 years of age and younger, and older women, with breast cancer.

A fundamental difference in the pathophysiology is that young women tend to present with a more advanced stage of breast cancer than do older women, and, hence, the earliest possible detection is crucial to their optimal prognosis. It is important to emphasize that a malignant breast neoplasm which has grown to a palpable size usually presents as a painless mass [2]. Many studies have shown that women who regularly practice breast self-examination initially present with smaller tumors and with neoplasms that less frequently involve the axillary lymph nodes [3–10]. Despite these facts, the American Cancer Society decided to change their previous guideline for regular breast self-examination (BSE), recommending instead that BSE now be optional because benefits of performing the self-examination had not been substantiated unequivocally in the literature [11].

The guidelines advocated by the American Cancer Society in 2003 for early breast cancer detection in asymptomatic women over 40 years of age were very specific: annual mammography, annual clinical breast examination, and an optional monthly breast self-examination. The guidelines are less stringent for females under 40 years of age and consist of a clinical breast examination every three years and an optional monthly breast self-examination. Mammographic screening in the patient population under 40 years of age remains controversial. The American Cancer Society eliminated its recommendation that all women perform monthly BSE after publication of studies such as the randomized trial of 266,062 women in Shanghai, which concluded that intensive instruction in BSE does not reduce the mortality rate of breast cancer [11]. Nonetheless, the society still recommends that women be informed of the potential benefits and limitations of BSE and that those women who wish to do so should receive instruction in proper BSE from their health care providers [11].

We hypothesized that younger women are more concerned with breast disease as it relates to their breast image and appearance, to their lifestyle, and to longer life expectancy. The various considerations and observations concerning young women and their breast disease led us to attempt to understand several concepts more clearly, including the relationships between breast carcinoma biology and age, breast carcinoma management patterns and age, and outcomes and age. 

The purpose of this study was to evaluate the characteristics that are unique to women 40 years of age and under, who present with breast cancer, in order to assess the role and potential benefits of BSE more meaningfully. Presumably, diagnosis and treatment of breast lesions are delayed in young women due to a low index of suspicion. Thus, we evaluated the detection, incidence, and treatment patterns of women 40 years of age and under with breast cancer who presented to our community teaching hospital over a 10-year period. The clinical and pathological characteristics of cancer in this patient population and the role of BSE as a screening tool in detecting malignancy in these patients were also examined.

## 2. Materials and Methods

Young women were defined as those 40 years old or younger. After approval by the Institutional Review Board, a retrospective review of a total of 822 women diagnosed with breast carcinoma during the decade between the years of 1994 and 2005 was completed at our community teaching hospital. Of those, 64 women were below 41 years of age, accounting for 7.7% of all breast cancers. Retrospective clinical information, which was obtained from the tumor registry, the hospital medical records, and office charts, was analyzed. In order to create a uniform basis for comparison, the tumors of all patients were restaged according to the TNM classification advocated by the American Joint Committee on Cancer (AJCC). Tumor size and the status of the axillary nodes were ascertained from review of the original pathology reports. Mann Whitney, Fisher Exact, and Chi-Squared tests were used as appropriate to compare the two age groups of breast cancer patients, and *P* < .05 was considered statistically significant. All of the 822 women with breast cancer were treated in our institution, and none were referred elsewhere. Surgical procedures were coded in accordance with the Data Acquisition Manual (DAM). Breast-conserving surgery was compared with mastectomy in both age groups.

## 3. Results

Throughout the decade-long study period, a statistically significant increase in age-adjusted incidence of breast cancer among women 40 years of age and younger became obvious (*P* < .005). The number of patients having a significant family history for breast cancer in both groups (>40 years and <41 years) could not be derived accurately from the family history data recorded on the charts, especially during the 1990s. The entries for these data improved remarkably in the 21st century but were sufficiently inconsistent to be of significant value in this group of patients. Similarly, the situation was comparable regarding genetic testing in these patients during the time period of the study (1994–2005). Of the 758 older patients, over the age of 40, three hundred eighty two (49%) discovered their cancer by mammography, 278 (39%) by self-examination, and 98 (14%) by a clinical breast examination. Magnetic resonance imaging (MRI) was not available for use in the diagnosis of patients with breast cancer during the decade of this study. Estrogen and progesterone receptor status was known in 660 (80%) patients and unknown in 166 (20%). Lymph nodes were positive in 156 (21%) patients and unknown in 154 (21%) of the older patients. At the time the older patients had their breast cancers diagnosed, 122 (16%) patients had Stage 0 disease, 331 (44%) had Stage I, 212 (28%) had Stage II, 48 (6%) had Stage III, 21 (3%) had Stage IV, and 24 (3%) had an indeterminate stage. Of 822 women diagnosed with breast cancer in our hospital from 1994 to 2004, sixty four (7.7%) were 40 years of age or younger. The ages of the 64 young patients ranged from 19 to 40 years, with a mean age of 36.4 years. Forty four (69%) of these younger women discovered their breast cancers by breast self-examination, 17 (18%) by mammography, and 3 (5%) by professional clinical breast examination. This series spans 10 years, and the surgical procedures chosen reflect the dominant influences of that time period. Thirty three (50%) of these young patients underwent a modified radical mastectomy, and 2 (3%) underwent a simple mastectomy. Only 28 (44%) of the patients chose to have a breast conservation procedure. The remaining three patients had no surgical treatment recorded. Hormone receptors were positive in 41 (64%) patients. Lymph nodes were positive for malignancy in 27 (42%) of patients sampled. There were 14 (22%) patients with Stage 0, 14 (22%) patients with Stage I disease, 25 (39%) patients with Stage II, 9 (14%) with Stage III, 0 with Stage IV, and 2 (3%) young patients with an unknown stage. Six of the patients studied died of their disease. The number of young patients diagnosed per year appeared to be increasing, even in this relatively small cadre, as follows: 5 (1994), 5 (1995), 2 (1996), 3 (1997), 6 (1998), 7 (1999), 6 (2000), 8 (2001), 6 (2002), 6 (2003), and 10 (2004). 

When compared with women under 41 years of age, a higher percentage of women over 40 years of age discovered their breast cancers by mammography (49%) and had a higher number of hormone sensitive tumors (87%). The frequency of lymph node metastases reported in the older women was one-half that in the younger women (21% versus 42%), and a higher percentage of younger women presented with more advanced disease, a difference which was statistically significant (*P* < .05). Many of the patients (in the unknown lymph node status category) had undergone simple mastectomy in the 1990s, thus accounting for the overall 20% “unknown” lymph node status, which was 20% in the >40 year-old and 19% in the <41 year-old group (Table 1).

## 4. Discussion

Carcinoma of the breast in young women is a relatively uncommon occurrence, with our data depicting an incidence of approximately 8% of all women with breast cancer. This correlates with findings of other authors who have reported incidences of 0.3% to 25% [1, 12–14]. Mammography remains the premier screening tool for older women; however, it is of questionable value in young women primarily because small lesions tend to be undetectable or interpreted as benign secondary to the increased density of the breasts of young women, which renders radiographic differentiation of normal from abnormal tissue difficult [15]. Ashley and colleagues found that 77% of mammograms in young women produced an X-ray suspicious for malignancy, and only 23% were obviously benign [16]. Ultrasonography identified the pathology correctly in 58%, while fine-needle aspiration (FNA) cytology was most precise, identifying 78% of tumors as definitely malignant and 15% as suspicious, for an overall accuracy of 93%. The most frequent lesion noted in this cadre of patients, as well as in other series, is infiltrating duct carcinoma [17].

The prognosis of women with cancer of the breast is related to a variety of clinical findings and pathological factors, especially the status of the axillary lymph nodes and the tumor size. Studies have also shown that young women with breast cancer have a lower 5-year relative survival rate than older women [12–14, 18, 19]. It has been postulated that the tumors in younger women are more aggressive and less responsive to hormone therapy and that these characteristics account for the differences in 5-year survival between the age groups [20] (Table 2).

However, it remains unclear whether the dismal outcome in the younger age group is a reflection of more advanced disease at the time of diagnosis and/or is due to a difference in underlying tumor biology [12, 14, 15, 18]. Finally, it has been observed commonly that breast carcinoma appears to be more indolent generally in older women, and most particularly in the elderly [21].

## 5. Conclusion

In 2003, the American Cancer Society recommended that breast self-examination be optional primarily because of a lack of unequivocal supporting evidence of benefit. Hence, the only screening guideline currently suggested for women 18–40 years of age is a clinical breast examination every three years. However, 5-year survival among young women diagnosed with breast cancer is clearly related to the stage of the disease at the time of diagnosis [17, 22]. Thus, early diagnosis and treatment of breast carcinoma are essential for optimal prognosis. Breast self-examination has not been encouraged or recommended, in part, because it has not been consistently associated with a decrease in morbidity or mortality, and because it purportedly can cause increased patient anxiety, especially when biopsies of breast masses that are discovered on self-examination are performed. We propose that the anxiety can be dispelled or ameliorated by quality time spent properly counseling and supporting the patient. 

Our data confirm that young women with breast cancer are likely to present with a more advanced stage of the disease compared with older patients. In response to increasing breast cancer in young women under 41 years of age in recent years, further investigation focused on this age group is indicated, and encouragement of proper breast self-examination is warranted and should be advocated as a quality health maintenance practice.

##  Conflict of Interests

None of the authors have any conflict of interests to disclose.

## Figures and Tables

**Table 1:** Data comparing younger and older breast cancer patients.

	Total patient number (*N*)	%	*N* > 40 years (old)	%	*N* < 41 years (young)	%	*P* value
	822		758	92	64	8	

Method of discovery							
Mammography	399	49	382	49	17	18	
Self-breast examination	322	39	278	37	44	69	
Clinical examination	111	14	98	13	3	5	<.0001

Lymph node status							
Lymph nodes positive	183	23	156	21	27	42	
Lymph nodes negative	473	57	448	59	25	39	.01
Lymph nodes unknown	166	20	154	20	12	19	

ER/PR receptor status							
ER/PR receptor positive	660	80	619	82	41	64	
ER/PR receptor negative	162	20	139	18	23	36	.01

Stage of cancer		17					
Stage 0	136		122	16	14	22	
Stage I	345	42	331	44	14	22	
Stage II	237	29	212	28	25	39	
Stage III	57	7	48	6	9	14	
Stage IV	21	3	21	3	0	0	<.005
Stage unknown	26	3	24	3	2	3	

Treatment							
Lumpectomy	651	79	623	76	28	44	
Simple mastectomy	16	2	14	2	2	3	
MRM	147	18	117	15	33	50	
No surgical treatment	8	1	5	0.7	1	1	<.05

**Table 2:** Five-year survival in breast cancer patients according to age.

Patient age at diagnosis	5-year survival percentage
Women age 40 years and younger	82%
Women age 41–74 years	89%
Women age 75 years and over	88%
